# Copper(II) Methacrylate Complexes with Imidazole Derivatives—Structural, Spectral and Antitumor Features

**DOI:** 10.3390/molecules29174010

**Published:** 2024-08-24

**Authors:** Dragoș Vlad Teodoru, Rodica Olar, Cătălin Maxim, Mihaela Bacalum, Mina Răileanu, Emilia-Elena Iorgulescu, Gina Vasile Scăețeanu, Mihaela Badea

**Affiliations:** 1 Department of Inorganic and Organic Chemistry, Biochemistry and Catalysis, Faculty of Chemistry, University of Bucharest, 90–92 Panduri Str., 050663 Bucharest, Romaniarodica.olar@chimie.unibuc.ro (R.O.); catalin.maxim@chimie.unibuc.ro (C.M.); 2 Department of Life and Environmental Physics, Horia Hulubei National Institute for Physics and Nuclear Engineering, 30 Reactorului Str., 077125 Măgurele, Romania; bmihaela@nipne.ro (M.B.); mina.raileanu@nipne.ro (M.R.); 3 Department of Analytical Chemistry, Faculty of Chemistry, University of Bucharest, 90–92 Panduri Str., 050663 Bucharest, Romania; 4 Department of Soil Sciences, Faculty of Agriculture, University of Agronomic Sciences and Veterinary Medicine, 59 Mărăști Str., 011464 Bucharest, Romania; gina.scaeteanu@agro-bucuresti.ro

**Keywords:** copper complex, crystal structure, imidazole derivative, methacrylate, mouse melanoma cells

## Abstract

A series of five novel copper(II) complexes with imidazole derivatives having general core Cu(R-Im)_2_(Macr)_2_ (Macr = methacrylate anion; R-Im = 2-methylimidazole/2-MeIm, 4-methylimidazole/4-MeIm, 2-ethylimidazole/2-EtIm, 2-isopropylimidazole/2-*i*PrIm) has been synthesized and characterized by elemental analysis, Fourier Transform Infrared spectroscopy (FTIR), electronic reflectance spectroscopy, cyclic voltammetry, thermal analysis and single crystal X-ray diffraction. All complexes crystalize in a monoclinic crystal system and form a complex supramolecular network developed through hydrogen bonds. The stereochemistry of the copper ion is distorted octahedral except for the compound with 4-methylimidazole for which the geometry is square-pyramidal. The imidazole derivatives act as unidentate while methacrylate ions are chelated except for compound with 4-methylimidazole where is unidentate. All ligands and complexes inhibited B16 murine melanoma cells in a micromolar range, but the complex with 2-isopropylimidazole was more active. Furthermore, all species do not affect the healthy BJ cells in the concentration range used for assays.

## 1. Introduction 

Copper is an essential trace element present in many life systems. It is incorporated into various proteins with antioxidant activity [1], catalytic properties [2] or involved in oxygen transport [3].

Furthermore, copper complexes, in particular those with carboxylate and N-heterocyclic ligands, are recognized due to valuable properties and/or interesting structural features. So far, copper complexes with carboxylate and imidazole derivatives as ligands were reported for their anticonvulsant [4,5], antidiabetic [6], and good antitumor [7] activities as well as a model for DNA interaction to obtain chemotherapeutic agents [8,9]. The catalytic activity of copper complexes with carboxylate and N-donor ligands were also reported [10,11].

Many research studies showed significant progress in the utilization of copper complexes as antibacterial agents with satisfactory results [11,12].

Complexes [Cu(1,2-Me_2_HIm)_2_(Hsal)_2_] and [Cu(1,2-Me_2_HIm)_3_(sal)] (Me_2_HIm = dimethylimidazole, H_2_sal = salicylic acid) have proven high SOD-like activities and consequently may act as good mimics for the native Cu, Zn-SOD enzyme [13].

Concerning structural varieties of copper(II) carboxylate complexes with imidazole derivatives, there are reported mononuclear [8,9,11,12,13,14,15,16,17,18], binuclear [18,19], trinuclear [20,21] or polynuclear compounds [22]. Even though the great versatility of carboxylate ligands is evidenced in many complexes, the trinuclear mixed valence copper (I, II) complex [Cu_3_(Macr)_5_(HIm)_3_(H_2_O)], (HMacr = methacrylic acid, HIm = imidazole) showed two coordination modes for methacrylate anions (μ_2_-methacrylate-O,O’ bridge and unidentate) [20]. In contrast, for complex [Cu(HIm)_4_](1,4-bdc) (H_2_bdc = benzenedicarboxylic acid), carboxylate anions are in the outer coordination sphere and are linked through electrostatic forces and hydrogen bonds [23].

A promising category of antitumor agents is represented by copper complexes, based on their ability to inhibit tumor growth. The better results have been achieved by combining chemotherapy with additional methods such as immunotherapy or gene therapy [24]. Some of the advantages of using copper complexes as antitumor drugs are price affordability [25] and reduced systemic toxicity in comparison with platinum-based compounds usually used in chemotherapy [26].

For instance, complexes [Cu(bz)_2_(2-R-HIm)_2_] (Hbz = benzoic acid, 2-R-HIm = imidazole/2-methylimidazole) revealed significant inhibition activity on human liver hepatocellular carcinoma (HepG2) cell lines [9]. An evaluation of antitumor properties of a plethora of copper complexes with carboxylate and benzimidazole was reported [27]. Furthermore, a copper complex with mixed ligands, with the anions of 4-methylcoumarin-6,7-dioxyacyeic acid (4-Mecdoa) and 1,10-phenantroline (phen), [Cu(4-Mecdoa)(phen)_2_], has proven its activity against kidney (A-498) and liver (Hep-G2) cancer cells, with the activity being more potent than that of cisplatin [28].

Copper complexes [Cu(1,2/1,3/1,4-bdc)(phen)_2_] have been reported due to higher antitumor activity against breast (MCF-7), prostate (DU145) and colon (HT29) cancer cells in comparison with cisplatin [29]. Also, a dicopper(II) cation, [Cu_2_(μ-1,4-bdc)(phen)_4_]^2+^ exhibits in vitro cytotoxicity towards human-derived ovarian cancer cell line (SK-OV-3), which is resistant to cisplatin [30]. Other copper complexes, [Cu(bzac)(phen)X] (Hbzac = 1-phenyl-1,3-butanedione, X = NO_3_ or ClO_4_), were reported as potential therapeutic species for melanoma [31].

In the context of our previous research concerning synthesis and full characterization of mixed complexes with carboxylate ions and imidazole derivatives [32,33,34], and, considering the proven antitumor potential of similar copper(II) complexes, we have explored the possibility of combining methacrylate anions and imidazole derivatives with this ion to obtain different species with potential antitumoral activity. Our previous research revealed an interesting chemistry of complexes containing acrylate/methacrylate ions and imidazole/benzimidazole derivatives. This statement refers to the various coordination modes of carboxylate ions, the possibility of obtaining multiple compounds from the same synthesis and the formation of geometric isomers [32,33,34,35]. On the other hand, these complexes have shown promising biological applications as antimicrobial and/or antitumor agents.

The following five new complexes were obtained and characterized: [Cu(2-MeIm)_2_(Macr)_2_] (**1**), [Cu(4-MeIm)_2_(Macr)_2_(H_2_O)] (**2**), [Cu(2-EtIm)_2_(Macr)_2_] (**3**), [Cu(2-EtIm)_2_(Macr)_2_]·H_2_O (**4**) and [Cu(2-*i*PrIm)_2_(Macr)_2_]·CH_3_OH (**5**), where Macr is methacrylate anion, 2-MeIm, 4-MeIm, 2-EtIm and 2-*i*PrIm is 2-methyl-, 4-methyl-, 2-ethyl- and 2-isopropyl-imidazole, respectively.

## 2. Results and Discussion

**Scheme 1:**
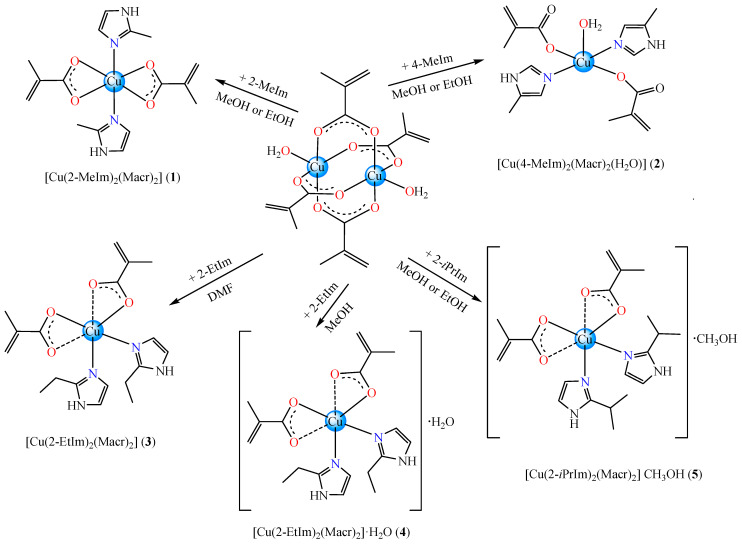
Synthetic route for complexes.

Complexes (**1**)–(**5**) were obtained from the direct reaction of copper methacrylate with imidazole derivatives in 1:2 molar ratio by using different solvents (methanol, ethanol, DMF) followed by slow evaporation at room temperature as depicted in Scheme 1. All complexes are soluble in DMF and DMSO.

### 2.1. Description of the X-ray Crystal Structures of the Complexes

All complexes were characterized by single crystal X-ray diffraction. A summary of crystallographic data is presented in Supplementary Table S1, while a selection of bond lengths and angles is displayed in Supplementary Tables S2 and S3.

**Figure 1:**
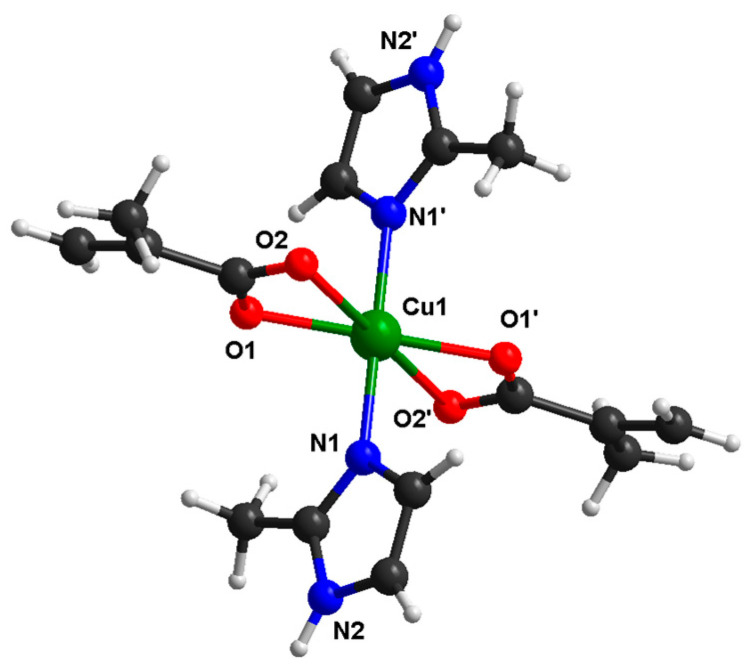
Mononuclear unit in (**1**) together with the numbering scheme. Color legend: green, Cu; gray-80%, C; red, O; blue, N; gray-25%, H. ′ = 1 − *x*, 1 − *y*, 1 − *z*.

The compound [Cu(2-MeIm)_2_(Macr)_2_] (**1**) was obtained as crystalline material in the monoclinic, *P*2_1_/n space group. The asymmetric unit has one-half of a copper(II) ion lying about an inversion centre with one anionic methacrylate and one 2-MeIm molecule in apical positions (Figure 1). For the mononuclear (**1**) moiety, the metal centres present a slightly distorted octahedral geometry, with a continuous shape measure (CShM) value of 8.397 (Table S4). The copper(II) ion presents an O4N2 environment resulting from two coordinated carboxylate anions and two nitrogen atoms from the imidazole ligand. The Cu-O distances vary between 1.9713(14) Å and 2.671 Å for methacrylate anions and Cu1–N1 = 1.9891(14) Å for imidazole ligand, values typical for the Cu(II) ion.

**Figure 2:**
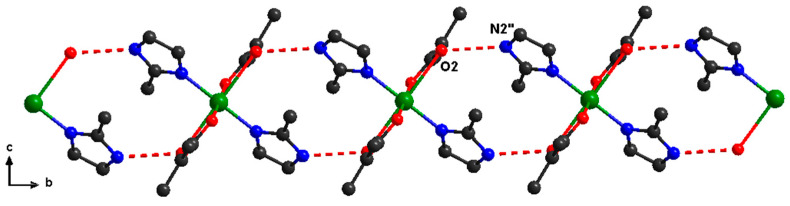
Supramolecular chains in (**1**). Color legend: green, Cu; gray-80%, C; red, O; blue, N; gray-25%, H.″ = *x*, 1 + *y*, *z*.

At the supramolecular level, the mononuclear units interact through hydrogen bonds established between uncoordinated nitrogen of 2-methylimidazole and oxygen atoms from carboxylate ligands (O2–N2″ = 2.782 Å). The resulting supramolecular chains are running along the *b* crystallographic axis (Figure 2).

**Figure 3:**
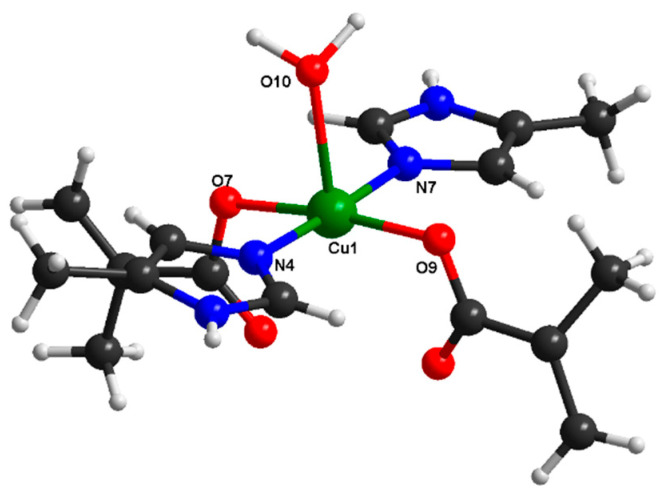
Molecular structure of the complex [Cu(4-MeIm)_2_(Macr)_2_(H_2_O)] (**2**). Color legend: green, Cu; gray-80%, C; red, O; blue, N; gray-25%, H.

The reaction between copper methacrylate and 4-MeIm affords a mononuclear complex, [Cu(4-MeIm)_2_(Macr)_2_(H_2_O)] (**2**), with two 4-MeIm molecules coordinated to each copper ion. The crystallographic investigation of (**2**) reveals a neutral centrosymmetric complex (Figure 3). Each copper(II) ion is pentacoordinated with a square-pyramidal geometry. Two carboxylato oxygen and two nitrogen atoms (from the 4-MeIm ligands) describe the basal plane (Cu1 − O9 = 1.992(11), Cu1 − O7 = 1.998(10), Cu1 − N7 = 1.978(13), Cu1 − N4 = 1.978(14) Å, a = 1 − *x*, −*y*, 1 − *z*), with an oxygen atom arising from a water molecule in the apical position (Cu1 − O10 = 2.400(8) Å). The distance between the copper ions within the binuclear entity is 6.532 Å.

**Figure 4:**
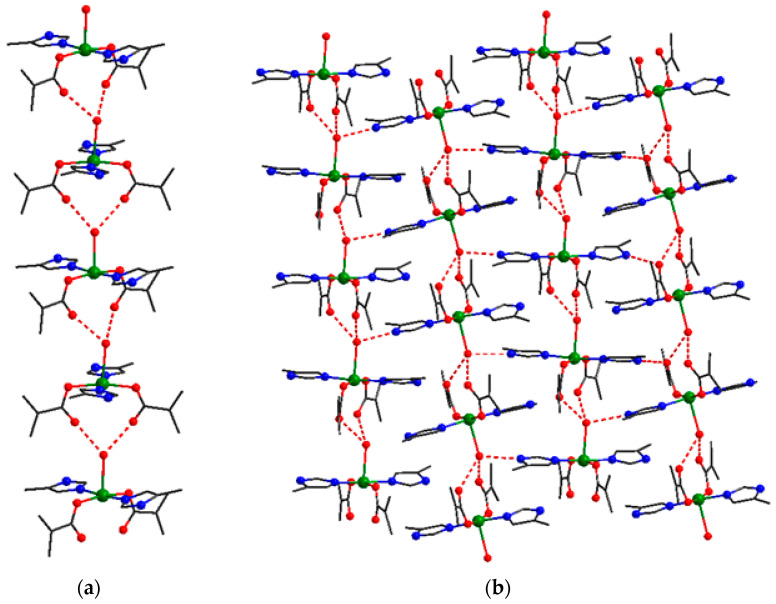
View of a 1D motif in crystal (**2**) (**a**) and perspective view of the layers resulted by connecting the chains by imidazol bridges (**b**). Color legend: green, Cu; gray-80%, C; red, O; blue, N; gray-25%, H.

The analysis of the packing diagram reveals the formation of supramolecular chains, though intermolecular hydrogen bond interactions (oxygen-oxygen distances vary between 3.46 and 3.83 Å) established between water ligands and uncoordinated oxygen atoms (Figure 4a). Further, the monodimensional structure is expanded in a second direction by hydrogen bonds which implies the uncoordinated nitrogen atoms from the imidazole derivative (Figure 4b).

**Figure 5:**
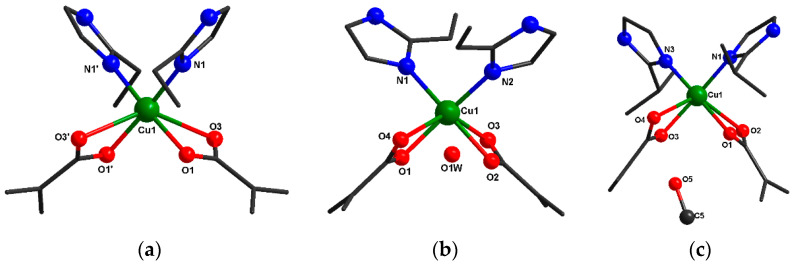
Crystal structure of the complexes (**3**) (a), (**4**) (b) and (**5**) (c). Color legend: green, Cu; gray-80%, C; red, O; blue, N; gray-25%, H. ′ = − *x*, *y*, 1/2 − *z*.

The crystallographic analysis for compounds [Cu(2-EtIm)_2_(Macr)_2_] (**3**), [Cu(2-EtIm)_2_(Macr)_2_]·H_2_O (**4**) and [Cu(2-*i*PrIm)_2_(Macr)_2_]·CH_3_OH (**5**) reveals the presence of similar structures with two imidazole derivatives as ligands coordinated in the *cis* position to metal ions together with two chelate methacrylato ions (Figure 5a–c). The copper(II) centres present a slightly distorted octahedral geometry, with a continuous shape measure (CShM) value of 8.207 for (**3**), 7.239 for (**4**) and 8.176 for (**5**) (Supplementary Table S4).

The difference between these structures is the presence of crystallizations molecules (water or methanol) which will influence the resulting supramolecular structures.

**Figure 6:**
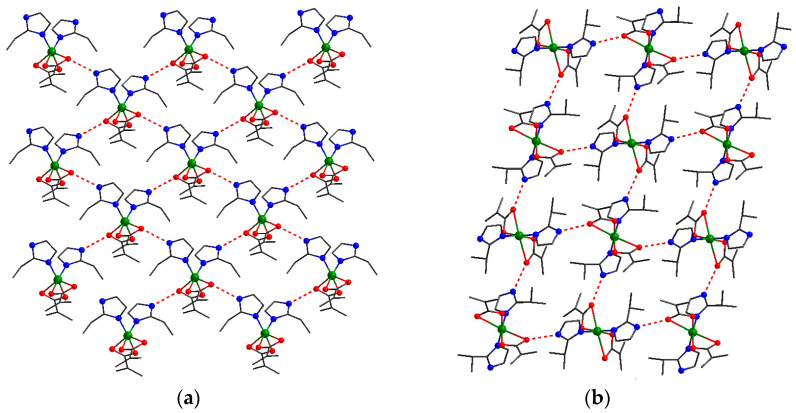
Supramolecular 2D structures in (**3**) (**a**) and (**5**) (**b**). Color legend: green, Cu; gray-80%, C; red, O; blue, N; gray-25%, H.

At the supramolecular level, for compounds (**3**) (Figure 6a) and (**5**) (Figure 6b), there was the formation of a bidimensional structure built up by hydrogen bond interactions between the uncoordinated nitrogen atoms and oxygen atoms arising from methacrylato ligands. The presence of water molecules in (**4**), increases the dimensionality of the network, resulting an extended hydrogen bonds supramolecular structure (Supplementary Figure S1).

A comparison of the Cu(II) complexes with the same imidazole derivatives and acrylate ions [32] reveals differences concerning the number and nature of isolated species. Thus, from the copper acrylate reaction with 2-methylimidazole and 2-ethylimidazole, both *cis* and *trans* isomers were obtained, while for methacrylate, only one geometric isomer was isolated, *trans* for 2-MeIm and *cis* for 2-EtIm.

Concerning the stereochemistry and carboxylate coordination mode, for both series of complexes, an octahedral surrounding and chelate behavior of acrylate and methacrylate were observed. Also, a common characteristic of these systems exists in the isolation of several compounds with different composition and/or stereoisomerism [32,35].

### 2.2. Characterization of Complexes

#### 2.2.1. Fourier Transform Infrared Spectroscopy

The most important bands in the FTIR spectra of complexes and their corresponding assignments are summarized in Table 1. All spectra (Supplementary Figure S2) contain bands characteristic for both ligands. Thus, in the ranges of 3130–3175, 1570–1650 and 740–1300 cm^−1^, several bands appear with different intensities that can be assigned to combined vibrational modes for the imidazole ring [32,36]. The band around 1645 cm^−1^ assigned to the stretching mode for imidazole C=N group is shifted to lower wavenumbers in comparison with free imidazole derivatives. This shift is indicative of the involvement of the nitrogen atom in coordination. The characteristic bands for imidazole ligands are presented in Supplementary Table S5.

The characteristic bands for the carboxylate group can be noticed in the range of 1365–1580 cm^−1^. Comparing the differences between wavenumbers corresponding to the two stretching vibration modes of this group, Δ [Δ = ν_as_(COO) − ν_s_(COO)], with that of 136 cm^−1^ characteristic for free methacrylate, it is obvious that for complex (**2**) a value of 184 cm^−1^ is in accordance with an unidentate coordination mode, while one ranging between 115–134 cm^−1^ for the other species of the series comes from a bidentate coordination mode of this anion [37,38].

The wide band at 3311 and 3441 cm^−1^ in the spectra for complexes (**2**) and (**4**) was assigned to stretching vibration mode for water molecules while that found at 3450 cm^−1^ for compound (**5**) comes from the stretching vibration mode of the OH group from methanol [39].

#### 2.2.2. Electronic Spectroscopy

**Figure 7:**
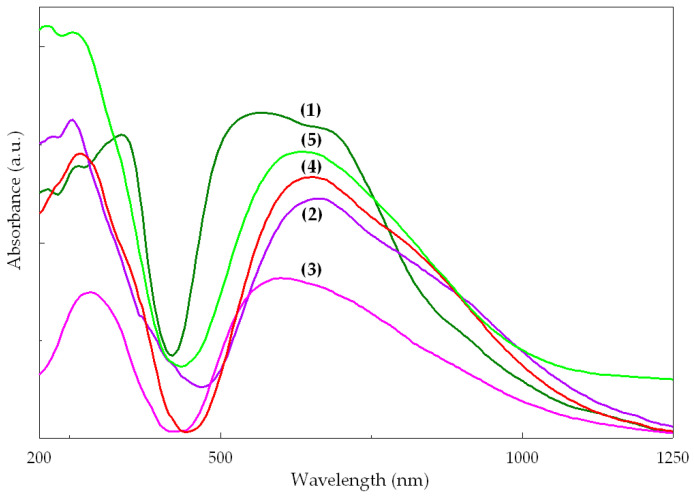
Electronic spectra of complexes.

The electronic spectra of the complexes are shown in Figure 7. The band assignments (Supplementary Table S6) were made, according to the literature, for a square pyramidal stereochemistry for compound (**2**) and for a distorted octahedral stereochemistry for the other compounds [40].

Considering that the antitumor assay was run in DMSO solutions, the stability of the complexes in this solvent was examined using the same technique. It is worth mentioning that no significant changes were detected, suggesting that all compound solutions remain stable for at least 48 h (Supplementary Figure S3). The solution spectra are similar for all complexes, exhibiting the pattern of square-planar stereochemistry. Furthermore, the absorption maxima are shifted to higher wavelengths in all cases. This shift may arise from the coordination of DMSO accompanied by the change in the coordination mode of the methacrylate.

#### 2.2.3. Voltammetric Studies

The redox properties of the complexes were studied by cyclic voltammetry (CV) in the cathodic range by scanning the potentials between +0.60 ÷ −1.80 V to obtain information concerning their reduction potentials, at 0.05 V/s scan rate (Supplementary Figure S4).

A comparison of the CV peak potentials of the complexes with that of the copper methacrylate (Cu(Macr)_2_·H_2_O) under the identical experimental conditions are presented in Table 2. From data presented in this table, it is observed that all complexes display two more or less well defined successive reduction peaks, that can be assigned to Cu(II)/Cu(I)/Cu(0). For complexes (**1**), (**2**) and (**4**), the reduction peaks are well defined, which means that the ligands stabilized the ion Cu(I). It is also observed that the peak potentials shift to cathodic values for both the first and the second reduction peak and, on anodic branch of the voltammograms, the slightly sharp shape of the first oxidation peak indicates reoxidation of the metallic copper deposited on the electrode surface, confirming the Cu(I)/Cu(0) reduction step.

For complexes (**3**) and (**5**), the reduction peaks present at more negative potentials, are less defined. This electrochemical behaviour can be explained by the formation of bidimensional structures at supramolecular level. The cyclic voltammograms recorded under the same experimental conditions for the 1 mM solution of ligands in DMSO, present on the cathodic range, a peak in the range of −0.719 ÷ −0.870 V vs. Ag/AgCl/0.1 M Bu_4_NClO_4_ solution in DMSO and a corresponding oxidation at −0.830 ÷ −0.870 V.

#### 2.2.4. Thermal Behaviour

Thermal degradation of the complexes provides insight into their stability and confirms the presence of solvents and metal content. From the data presented in Table 3, it can be observed that all complexes melt, and after solvent elimination, the decomposition follows two overlapping steps corresponding to the release of imidazole derivatives and the oxidation of methacrylate ions.

### 2.3. Antitumor Assay

Copper as an essential trace element is also studied to develop nonplatinum anticancer metallodrugs. Copper complexes are biocompatible, exhibit fewer adverse effects, have a wide therapeutic window, act by inhibiting cancer via multiple pathways, and are sometimes more potent than cisplatin and exhibit activity on cisplatin resistant tumor cells [41,42,43].

Hence, in the continuous search for new drugs with lower toxicity and reduced side effects, Cu(II) complexes are preferred, including for melanoma chemotherapy. The studies in the field, provided species developed with N-heterocycle-based ligands such as imidazole [27,31,44], 1,10-phenantroline [45], or triazolopyrimidine [46] derivatives which are active in the micromolar range on B16 melanoma cell lines.

**Figure 8:**
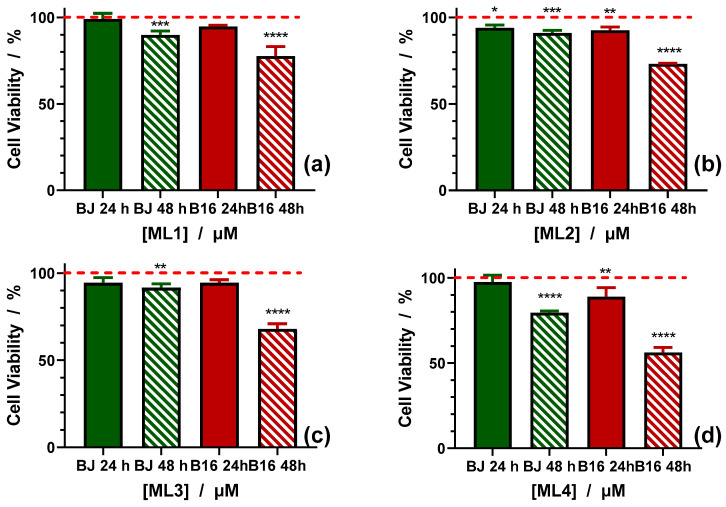
Cell viability induced by 100 µM of the four imidazole derivatives (ML1 = 2-MeIm (**a**); ML2 = 4-MeIm (**b**); ML3 = 2-EtIm (**c**); ML4 = 2-*i*PrIm (**d**)) against BJ and B16 cells treated for 24 and 48 h. At least three independent experiments are performed and data are represented as the means ±  SD. *p* values based on ANOVA analysis with Bonferroni’s comparison post-test versus control condition: * *p* < 0.05, ** *p* < 0.01, *** *p* < 0.001, **** *p* < 0.0001.

In view of the above data, the new copper(II) complexes were assayed on B16 mouse melanoma cells in comparison with BJ human fibroblast cells. The two cell line viability was investigated at 24 and 48 h, first for the four ligands (2-MeIm, 4-MeIm, 2-EtIm and 2-*i*PrIm) used to obtain the five studied complexes. The curves are presented in Figure 8 and Supplementary Figure S5. The results show that BJ cell viability is not affected when cells are treated for 24 and 48 h independent of the time and concentrations with the first three compounds (2-MeIm, 4-MeIm and 2-EtIm). However, the viability of BJ cells is affected when treated with 2-*i*PrIm for 48 h at concentrations higher than 6.25 μM (Supplementary Figure S5d). The cell viability decreases to 79% when cells are treated with 100 μM (Figure 8).

When treated with these ligands, melanoma cells are more effected than BJ cells especially when treated for 48 h, at concentrations higher than 6.25 μM. For 2-MeIm, cell viability decreases at the highest concentration to 77.63%, for 4-MeIm to 73.18%, for 2-EtIm to 67.94% and for 2-*i*PrIm to 56.26% (Figure 8). The results indicate that the four ligands show an increased toxicity against melanoma B16 cells, and almost no toxicity for normal BJ cells. Surprisingly, the changes in imidazole substituents from methyl to isopropyl also lead to an increase of toxicity against B16 cells.

**Figure 9:**
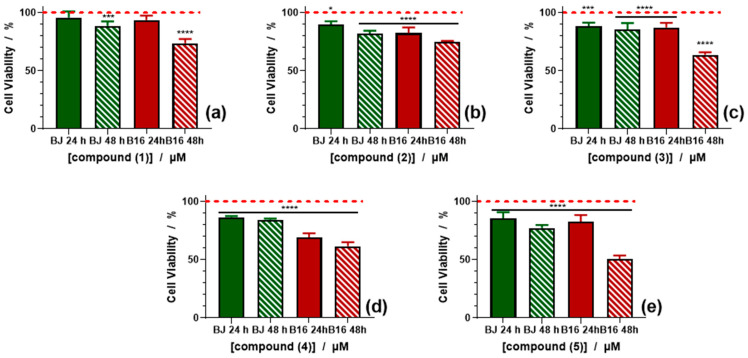
Cell viability induced by 100 µM of the complexes (**1**) (**a**), (**2**) (**b**), (**3**) (**c**), (**4**) (**d**) and (**5**) (**e**) against BJ and B16 cells treated for 24 and 48 h. At least three independent experiments are performed and data are represented as the means ± SD. *p* values based on ANOVA analysis with Bonferroni’s comparison post-test versus control condition: * *p* <0.05, *** *p* < 0.001, **** *p* < 0.0001.

Furthermore, the five complexes were also tested in similar conditions as the ligands reported above and the curves are presented in Figure 9 and Supplementary Figure S6. As well as the ligands tested, the complexes have different effects on the normal cells compared with tumor cells.

Thus, for the normal BJ cells, the viability of the cells treated for 24 h with the five compounds dose not decrease below 85% at the highest concentration tested (Figure 9). When treated for 48 h, a slightly higher toxicity was found for BJ cells treated with compound (**4**) at 100 μM, where the viability decreased to 77%. All other compounds did not significantly affect the viability compared with the cells treated for 24 h.

When B16 cells were treated with the five compounds, an increase in toxicity over time was observed. For the cells treated for 24 h, the viability was not significantly different compared with the viability of BJ cells treated for 24 or 48 h. However, when treated for 48 h, the cell viability decreased at concentrations higher than 6.25 μM. There was also an increase of toxicity observed in compounds (**1**) to (**5**). Thus, compounds (**1**) and (**2**) exhibited the lowest toxicity, with a value around 73–74% at the highest concentration tested, followed by compounds (**3**) and (**4**), for which the concentration decreased around 61–63% and, finally, for compound (**5**), at the highest concentration tested, the viability decreased to 50% (Figure 9).

The results indicate that the new compounds have good antitumor activity. Previous imidazole Cu(II) compounds showed both antimicrobial [47,48] and antitumoral activity against various tumoral cells [47,49,50]. In their study, Morelli et al. investigated the antitumor effects of seven new copper complexes against several cell lines with encouraging results for one compound [50]. The concentration range which can lead to 100% cell growth inhibition is between 23 and 47 µM, values slightly smaller than the ones obtained in the same conditions for cisplatin. A different study has also reported cytotoxic effects against the MCF7 cell line in the range of 25–100 µM [49]. Alshehri et al. have synthesized three copper complexes which are 5–6 times more efficient against M-14 cells compared with cisplatin. This efficiency is due to their binding to DNA. A recent study by Gałczyńska et al. has showed the antifungal and toxic properties of new copper and cobalt complexes [47]. Similar to the compounds reported in this study, in the range of 7–250 µM, the Cu(II) and Co(II) complexes showed an increased toxicity towards the A549 tumour cell lines compared to the normal BEAS-2B cell line. Another study reported three compounds with both antimicrobial and antitumoral activity in the micromolar concentration range against HeLa and K562 cell lines [48].

Based on our results and compared with other Cu(II)-based compounds reported previously, we can conclude that the new compounds reported in this study show a good potential as antitumoral compounds. The most efficient one proved to be compound (**5**), which at the highest concentrations was able to reduce cell viability to half after 48 h of treatment.

A comparison with reported data concerning Cu(II) complexes with antimelanoma activity [27,31,44,45,46] indicated that those bearing benzimidazole or phenanthroline moieties are more a potent antimelanoma species, a characteristic linked to the enhanced intercalation abilities of this aromatic system [51]. As a result, the functionalisation of the imidazole ring with an aromatic nucleus could lead to copper complexes with better antitumor activity.

## 3. Materials and Methods

### 3.1. General Information

High purity reagents were purchased and used without further purification from Merk Schuchardt OHG (Hohenbrunn, Germany, acrylic acid), Fluka (Saint-Louis, MO, USA, CuCO_3_·Cu(OH)_2_), Sigma-Aldrich (Saint-Louis, MO, USA, imidazole derivatives).

The content of carbon, nitrogen and hydrogen has been determined using a PE 2400 analyzer (Perkin Elmer, Waltham, MA, USA). The FTIR spectra were recorded in KBr pellets with a Tensor 37 spectrometer (Bruker, Billerica, MA, USA) in the range of 400–4000 cm^−1^. Electronic spectra were recorded on solid samples (diffuse reflectance technique) in the range of 200–1250 nm, on a V670 spectrophotometer (Jasco, Easton, MD, USA) using Spectralon as the standard. The DMSO solution UV-VIS spectra were recorded on a Jasco V530 spectrophotometer (Jasco, Easton, MD, USA) in the range of 200–900 nm. The solution concentration for each complex was 10 μM. The thermal analysis (TG, DTG and DTA curves) was performed using a Labsys 1200 SETARAM instrument, alumina crucibles and samples of complexes with masses ranging from 15–20 mg. All measurements were conducted in synthetic air (flow rate 16.66 cm^3^ min^−1^) for a temperature range of 30–900 °C and heating rate of 10 °C min^−1^.

Cyclic voltammetry experiments were performed using a cell with a three-electrode configuration consisting of a platinum disk with 3 mm diameter as a working, Pt wire as the counter and Ag/AgCl separated from the solution by a bridge filled with a 0.1 M Bu_4_NClO_4_ solution in DMSO, as the reference electrode, against which all potentials reported are measured. The recording of cyclic voltammograms was performed using an Autolab PGSTAT 12 and the analysis was made by GPES 4.9 software. Prior to the experiment, the working electrodes were polished with 0.3 μm alumina powder and then rinsed with bi-distilled water before use and the solutions were purged with Argon (99.9999%) for 10 min. All the experiments were performed at room temperature. Tetrabutylammonium perchlorate (Bu_4_NClO_4_) 0.1 M was used as supporting electrolyte.

X-ray diffraction data for the crystals of compounds (**3**) and (**4**) were collected at 293 K on a STOE IPDS II diffractometer using a graphite-monochromator Mo Kα radiation source (λ = 0.71073 Å). For compounds (**1**), (**2**) and (**5**), data were collected at 293 K on a Rigaku XtaLAB Synergy, single source at offset/far and HyPix diffractometer using a graphite-monochromated Mo Kα radiation source (λ = 0.71073 Å). The structure was solved by direct methods and refined by using full-matrix least squares techniques based on *F^2^*. The non-H atoms were refined with anisotropic displacement parameters. Calculations were performed using the SHELX-2018 crystallographic software package http://shelx.uni-goettingen.de/. A summary of the crystallographic data and the structure refinement are presented in Supplementary Table S1. Crystallographic data (excluding structure factors) have been deposited with the Cambridge Crystallographic Data Centre with CCDC reference numbers 2,283,214, 2,283,215, 2,283,216, 2,283,217 and 2,283,218. These data can be obtained free of charge via http://www.ccdc.cam.ac.uk, or from the Cambridge Crystallographic Data Centre, 12 Union Road, Cambridge, CB2 1EZ, UK; fax: (+44) 1223-336-033; or e-mail: deposit@ccdc.cam.ac.uk, accessed on 1 June 2024.

### 3.2. Synthesis of Complexes

First, copper methacrylate was synthesized from basic copper(II) carbonate (5 g) and methacrylic acid (7 mL) in a methanolic solution. The mixture was stirred at room temperature for 8 h and then filtered off. From the resulting solution, after slow evaporation, green-blue crystals of copper methacrylate were obtained.

All complexes have been synthesized using a method similar to the following: the imidazole derivative (10 mmol) was added to a solution of copper methacrylate (5 mmol) in ethanol or methanol. The resulting solution was stirred at room temperature for 3 h. After slow evaporation at room temperature, the compounds (**1**), (**2**) and (**5**) crystallized. These were filtered off and washed with cold methanol.

From the system, copper(II) methacrylate-2-ethylimidazole, a mixture of blue and violet crystals was obtained (with more blue crystals of compound (**4**) typically obtained). These crystals can be separated mechanically. To obtain a predominant amount of violet crystals of compound (**3**), the synthesis was performed in DMF. Under these conditions, we succeeded in obtaining predominantly violet crystals.

[Cu(2-MeIm)_2_(Macr)_2_] **(1)** (dark blue single crystals); Anal. Calc. for CuC_16_H_22_N_4_O_4_: Cu, 15.97; C, 48.29; H, 5.57; N, 14.08; Found: Cu, 15.81; C, 48.19; H, 5.48; N, 14.15; yield 86% from methanol.

[Cu(4-MeIm)_2_(Macr)_2_(H_2_O)] (**2**) (dark blue single crystals); Anal. Calc. for CuC_16_H_24_N_4_O_5_: Cu, 15.28; C, 46.20; H, 5.82; N, 13.47; Found: Cu, 15.41; C, 46.13; H, 5.76; N, 13.53; yield 76% from methanol.

[Cu(2-EtIm)_2_(Macr)_2_] (**3**) (violet single crystals). Anal. Calc. for CuC_18_H_26_N_4_O_4_: Cu, 14.92; C, 50.75; H, 6.15; N, 13.15; Found: Cu, 15.01; C, 50.64; H, 6.08; N, 13.21; yield 58% from DMF, (~15% from ethanol or methanol).

[Cu(2-EtIm)_2_(Macr)_2_]·H_2_O (**4**) (blue single crystals). Anal. Calc. for CuC_18_H_28_N_4_O_5_: Cu, 14.31; C, 48.69; H, 6.36; N, 12.62; Found: Cu, 14.37; C, 48.54; H, 6.31; N, 12.68; yield 64% from methanol.

[Cu(2-*i*PrIm)_2_(Macr)_2_]·CH_3_OH (**5**) (dark blue single crystals); Anal. Calc. for CuC_21_H_34_N_4_O_5_: Cu, 13.07; C, 51.89; H, 7.05; N, 11.53; Found: Cu, 13.10; C, 51.74; H, 6.98; N, 11.58; yield 74% from methanol.

### 3.3. Cell Culture Conditions

The cells used in this study were human fibroblast cells (BJ—ATCC CRL-2522, Manassas, VA, USA) and mouse melanoma cells (B16—ATCC CRL-6475, Manassas, VA, USA) grown as described previously [37]. All cell cultivation media and reagents were purchased from Biochrom AG (Berlin, Germany) and Sigma-Aldrich (Darmstadt, Germany). The compounds were dissolved well in DMSO at a concentration of 100 mM and kept in the freezer between experiments. All the experiments were performed with a dilution of at least 1000 to have no effect from DMSO.

### 3.4. In Vitro Viability

Cell viability of the two cell lines was investigated using the MTT assay (3-(4,5-dimethylthiazol-2-yl)-2,5-diphenyltetrazolium bromide) as previously described [31]. Cells were treated with concentrations of 1 to 100 μM for 24 and 48 h. Following the treatment, cell viability was calculated and data represented using GraphPad Software 9 (Boston, MA, USA).

### 3.5. Statistical Analysis

The experiments were performed at least three times with at least 2 replicas per condition, per experiment. All data are presented as means ± standard deviations (SD), if not stated otherwise. The statistical analysis of the cell viability was carried out using the GraphPad Prism 5 software package (San Diego, CA, USA). One-way analysis of variance (ANOVA) was used to calculate statistical significance. A value of *p* < 0.05 was chosen to indicate that the difference is statistically significant.

## 4. Conclusions

Five new copper(II) complexes with mixed ligands, imidazole derivatives and methacrylate ions, were synthesized and structurally characterized. The mononuclear units of all complexes form a supramolecular network through hydrogen bond interactions between the uncoordinated nitrogen atoms of the imidazole derivatives and the oxygen atoms of the methacrylate ligands. All complexes demonstrated the ability to reduce the viability of B16 cells in a dose-dependent manner while showing no significant toxicity against BJ cells. The most potent compound, (**5**), was able to reduce cell viability to 50% after 48 h. Interestingly, the ligand 2-*i*PrIm encountered in complex (**5**) induced the highest decrease in melanoma B16 cell viability to 56.26% in comparison with the other imidazole derivatives. This behavior can be linked with isopropyl which is the most hydrophobic from substituents and will provide an increased lipophilicity for the ligand 2-iPrIm and complex (**5**), as well.

## Figures and Tables

**Table 1:** Infrared absorption bands (cm^−1^) for complexes.

(1)	(2)	(3)	(4)	(5)	Assignments
-	3311 m	-	3441 m	3450 m	ν(OH)
3173 m 3151 m 3133 m	3151 m 3129 m	3140 w 3059 w	3140 w 3060 w	3168 m 3155 m 3138 m	ν(CH) + ν(NH)
2960 m 2925 m	2954 m 2924 m	2969 w	2979 w	2970 m 2924 m	ν_as_(CH_2_)
2866 m	2891 m	2929 w	2930 w	2878 m	ν_s_(CH_2_)
1644 s	1644 s	1648 s	1645 s	1644 s	ν(C=N)
1577 m	overlapped	1613 vs	1605 vs	1581 m	δ(NH) + ν(C=C) + ν(C=N)
1547 vs	1590 vs	1549 vs	1549 vs	1547 vs	ν_as_(COO)
1413 m	1406 m	1434 m	1434 m	1420 m	ν_s_(COO)
1297 s	-	1260 w	1260 w	1294 s	δ(CH_2_)
1237 s	1236 m	1252 w	1257 w	1236 m	ν(C−N) + δ(CH)
1166 w	-	1163 w	1163 w	1158 w	ν(C−C) + ν(C−N) + δ(CH)
954 m	969 m	962 w	977 w	929 m	δ(CH) + δ(Im ring)
861 m	881 m	834 w	873 w	878 m	π(CH) + δ(Im ring)
749 m	780 w	763 w	768 w	760 m	π(CH)
631 m	625 m	618 w	618 w	623 w	δ(COO)

**Table 2:** Cyclic voltammetry data for Cu(II) methacrylate and complexes (**1**)–(**5**).

Compound	Epc1 *	Epc2 *	Epa1 *	Epa2 *	E_1/2_ (V) *^a^* Cu(I)/Cu(0)	E_1/2_ (V) *^b^* Cu(II)/Cu(I)
Cu(Macr)_2_·H_2_O		−0.598	−0.266	0.187		
(**1**)	−0.125	−0.760	−0.266	0.348	−0.563	+0.112
(**2**)	−0.215	−0.769	−0.185	0.419	−0.477	+0.102
(**3**)	−0.679	−1.031	−0.366	0.438	−0.699	−0.121
(**4**)	−0.296	−0.991	−0.145	0.388	−0.568	+0.040
(**5**)	−0.629	−1.051	−0.175	0.418	−0.613	−0.106

* All potentials are measured in V vs. Ag/AgCl/(0.1 Bu_4_NClO_4_ in DMSO); ***^a^*** Half-wave potentials were calculated for Cu(II)/Cu(0) using E_1/2_ = (E_pa1_ + E_pc2_)/2; ***^b^*** Half-wave potentials were calculated for Cu(II)/Cu(I) using E_1/2_ = (E_pa2_ + E_pc1_)/2.

**Table 3:** Thermal behavior data for complexes (**1**)**–**(**5**).

Complex	Step	Thermal Effect	Temp./°C	Δm_exp/_%	Δm_calc/_%	Process
[Cu(2-MeIm)_2_(Macr)_2_] (**1**)	1.	Endothermic	160	-	-	melting
	2.	Miscellaneous	160–282	41.7	41.3	-2(2-MeIm)
	3.	Exothermic	282–845	38.5	38.7	Macr oxidative degradation
	Residue (CuO)			19.8	20.0	
[Cu(4-MeIm)_2_(Macr)_2_(H_2_O)] (**2**)	1.	Endothermic	100	-	-	melting
	2.	Miscellaneous	120–280	39.8	39.5	-H_2_O, -2(4-MeIm)
	3.	Exothermic	280–900	36.5	37.1	Macr oxidative degradation
	Residue (CuO)			19.3	19.1	
[Cu(2-EtIm)_2_(Macr)_2_] (**3**)	1.	Endothermic	120	-	-	melting
	2.	Miscellaneous	140–290	45.5	45.1	-2(2-EtIm)
	3.	Exothermic	290–900	35.7	36.2	Macr oxidative degradation
	Residue (CuO)			18.8	18.7	
[Cu(2-EtIm)_2_(Macr)_2_]·H_2_O (**4**)	1.	Endothermic	80–125	4.2	4.1	-H_2_O
	2.	Endothermic	133	-	-	melting
	3.	Miscellaneous	145–300	43.9	43.3	-2(2-EtIm)
		Exothermic	300–900	33.9	34.7	Macr oxidative degradation
	Residue (CuO)			18.0	17.9	
[Cu(2-*i*PrIm)_2_(Macr)_2_]·CH_3_OH (**5**)	1.	Endothermic	155	-	-	melting
	2.	Endothermic	160–190	6.7	6.6	-CH_3_OH
	3.	Endothermic	190–380	45.3	45.5	-2(2-*i*PrIm)
	4.	Exothermic	390–900	31.6	31.6	Macr oxidative degradation
	Residue (CuO)			16.4	16.3	

## Data Availability

The original contributions presented in the study are included in the article/Supplementary Materials, further inquiries can be directed to the corresponding author/s.

## Supplementary Materials

The following supporting information can be downloaded at: https://www.mdpi.com/article/10.3390/molecules29174010/s1, Figure S1: 3D supramolecular structure in **4**, based on hydrogen bond interactions, Figure S2: FTIR spectra of complexes (**1**)–(**5**), Figure S3: Electronic spectra in DMSO solution of complexes (**1**)–(**5**): (blue—0 min, green—1 h, red—2 h, magenta—24 h, yellow—48 h), Figure S4: Cyclic voltammograms of copper complex (**1**)–(**5**); ((**1**)_—_(green line); (**2**)_—_(magenta line); (**3**)—(blue line); (**4**)_—_(light blue line); (**5**)—(black line)); Cyclic voltammogram for Cu(Mcr)_2_—red line, all concentrations 1 mM in DMSO; supporting electrolyte—0.1 M Bu_4_NClO_4_; scan rate: 0.050 V/s, working electrode, platinum disk, reference electrode, Ag/AgCl (0.1 M Bu_4_NClO_4_ in DMSO), Figure S5: Cell viability of the four imidazole derivatives (ML1 = 2-MeIm; ML2 = 4-MeIm; ML3 = 2-EtIm; ML4 = 2-*i*PrIm;) against BJ and B16 cells treated for 24 and 48 h. At least three independent experiments were performed and data are represented as the means ±  SD, Figure S6: Cell viability of the complexes (**1**) (a), (**2**) (b, (**3**) (c), (4) (**d**) and (**5**) (f) against BJ and B16 cells treated for 24 and 48 h. At least three independent experiments were performed and data are represented as the means ± SD, Table S1: Crystallographic data, details of data collection and structure refinement parameters for compounds (**1**)–(**5**), Table S2: Selected geometric parameters—bonds length (Å) in compounds (**1**)–(**5**), Table S3: Selected geometric parameters—angles (°) in compounds (**1**)–(**5**), Table S4: Continuous Shape Measures for the coordination polyhedron around the Cu(II), Table S5: Absorption maxima (cm^−1^) in FTIR spectra of imidazole derivatives, Table S6: Absorption maxima in UV-Vis-NIR spectra of complexes (**1**)–(**5**).
