# Supplementary material for: Copper(II) Methacrylate Complexes with Imidazole Derivatives—Structural, Spectral and Antitumor Features
Source: Molecules. 2024 Aug 24;29(17):4010. doi: 10.3390/molecules29174010 (PMC11396722; doi:10.3390/molecules29174010)
Supplement: Supplementary file 1 [file molecules-29-04010-s001.zip › molecules-3150090-supplementary.pdf]

Copper(II) methacrylate complexes with imidazole derivatives - structural, spectral and antitumor features

Dragoș Vlad Teodoru, Rodica Olar, Cătălin Maxim, Mihaela Bacalum, Mina Răileanu, Elena Emilia Iorgulescu, Gina Vasile Scăețeanu and Mihaela Badea

Table S1. Crystallographic data, details of data collection and structure refinement parameters for compounds (**1**) - (**5**).

Compound	(**1**)	(**2**)	(**3**)	(**4**)	(**5**)
Empirical formula	$C_{16}H_{22}CuN_4O_4$	$C_{16}H_{24}CuN_4O_5$	$C_{18}H_{26}CuN_4O_4$	$C_{18}H_{28}CuN_4O_5$	$C_{21}H_{34}CuN_4O_5$
Formula weight	397.91	415.93	425.98	443.99	486.06
Temperature/K	293(2)	293(2)	293(2)	293(2)	293(2)
Crystal system	*monoclinic*	*monoclinic*	*monoclinic*	*monoclinic*	*monoclinic*
Space group	$P2_1/n$	$P2_1$	C2/c	$P2_1/c$	$P2_1/c$
a/Å	7.8566(3)	13.0783(4)	11.9779(2)	9.1099(2)	10.6201(7)
b/Å	7.7160(2)	14.0622(4)	9.9997(3)	15.2947(3)	12.5292(6)
c/Å	15.5763(6)	33.6463(12)	18.2592(4)	15.6588(4)	18.7909(10)
α/°	90	90	90	90	90
β/°	100.643(4)	97.088(3)	102.006(5)	94.729(5)	99.827(6)
γ/°	90	90	90	90	90
Volume/$Å^3$	928.01(6)	6140.6(3)	2139.16(10)	2174.36(9)	2463.7(2)
Z	2	2	4	4	4
ρ_{calc}g/cm^3	1.424	1.348	1.323	1.356	1.310
μ/mm^{-1}	1.204	1.098	1.049	1.039	0.923
F(000)	414.0	2596.0	893.8	933.8	1028.0
Radiation	Mo Kα (λ = 0.71073)	Mo Kα (λ = 0.71073)	Mo Kα (λ = 0.71073)	Mo Kα (λ = 0.71073)	Mo Kα (λ = 0.71073)
Reflections collected	7717	19184	20311	22570	12945
Data/restraints/parameters	2301/0/117	19184/25/1435	1870/0/125	3835/2/262	4243/0/289
Goodness-of-fit on F^2	0.911	0.932	1.061	1.080	1.090
Final R indexes [I>=2σ (I)]	R_1 = 0.0317, wR_2 = 0.1068	R_1 = 0.0760, wR_2 = 0.2045	R_1 = 0.0409, wR_2 = 0.1029	R_1 = 0.0594, wR_2 = 0.1569	R_1 = 0.0445, wR_2 = 0.1168
Final R indexes [all data]	R_1 = 0.0405, wR_2 = 0.1180	R_1 = 0.1211, wR_2 = 0.2569	R_1 = 0.0433, wR_2 = 0.1062	R_1 = 0.0788, wR_2 = 0.1849	R_1 = 0.0523, wR_2 = 0.1211
Largest diff. peak/hole / e $Å^{-3}$	0.27/-0.40	1.09/-1.07	0.33/-0.39	0.66/-1.20	0.57/-0.44

Table S2. Selected geometric parameters - bonds length (Å) in compounds (**1**) - (**5**).

(**1**)			(**2**)			(**3**)			(**4**)			(**5**)		
Cu1	O1	1.9713(14)	Cu1	O7	1.998(10)	Cu1	O1	1.976(2)	Cu1	O1	1.977(3)	Cu1	O3	2.000(2)
Cu1	O1[1]	1.9713(14)	Cu1	O10	2.400(8)	Cu1	O1[2]	1.976(2)	Cu1	O2	2.009(3)	Cu1	O1	1.961(3)
Cu1	N1	1.9891(14)	Cu1	N4	1.978(14)	Cu1	N1	1.982(2)	Cu1	N1	1.994(4)	Cu1	N1	1.979(3)
Cu1	N1[1]	1.9891(14)	Cu1	O9	1.992(11)	Cu1	N1[2]	1.982(2)	Cu1	N2	1.977(4)	Cu1	N3	1.971(3)
O2	C5	1.252(2)	Cu1	N7	1.978(13)	O1	C1	1.249(4)	O1	C4	1.274(6)	O3	C6	1.269(4)

[1] = 1-*x*,1-*y*,1-*z*, [2] =-*x*,+*y*,1/2-*z*

Table S3. Selected geometric parameters - angles (°) in compounds (**1**) - (**5**).

(**1**)				(**2**)				(**3**)				(**4**)				(**5**)			
O1	Cu1	O1[1]	180.0	O7	Cu1	O10	93.2(4)	O1[2]	Cu1	O1	91.55(14)	O2	Cu1	O1	88.05(14)	O1	Cu1	O3	85.98(11)
O1[1]	Cu1	N1[1]	90.79(6)	O7	Cu1	O9	172.5(4)	N1	Cu1	O1[2]	173.98(10)	N1	Cu1	O1	91.46(15)	O1	Cu1	N1	91.73(11)
O1	Cu1	N1	90.80(6)	N4	Cu1	O7	88.4(5)	N1	Cu1	O1	89.73(10)	N1	Cu1	O2	162.74(14)	O1	Cu1	N3	169.86(11)
O1	Cu1	N1[1]	89.20(6)	N4	Cu1	O10	86.8(4)	N1[2]	Cu1	O1[2]	89.73(10)	N2	Cu1	O1	164.94(14)	N1	Cu1	O3	169.27(11)
O1[1]	Cu1	N1	89.20(6)	N4	Cu1	O9	91.7(5)	N1[2]	Cu1	O1	173.98(10)	N2	Cu1	O2	90.19(14)	N3	Cu1	O3	91.54(11)
N1[1]	Cu1	N1	180.0	O9	Cu1	O10	94.3(4)	N1[2]	Cu1	N1	89.60(14)	N2	Cu1	N1	94.60(15)	N3	Cu1	N1	92.46(12)
C5	O1	Cu1	107.59(12)	N7	Cu1	O7	90.4(5)	C1	O1	Cu1[2]	105.2(2)	C4	O1	Cu1	105.8(3)	C6	O3	Cu1	103.5(2)
C3	N1	Cu1	128.55(12)	N7	Cu1	O10	91.1(4)	C5	N1	Cu1	125.8(2)	C13	O2	Cu1	104.1(3)	C3	O1	Cu1	106.6(2)
C3	N1	C2	106.40(14)	N7	Cu1	N4	177.5(5)	C7	N1	Cu1	127.6(2)								
C2	N1	Cu1	124.97(11)	N7	Cu1	O9	89.7(5)												

[1] = 1-*x*,1-*y*,1-*z*, [2] =-*x*,+*y*,1/2-*z*

Table S4. Continuous Shape Measures for the coordination polyhedron around the Cu(II).

Geometry	(**1**)	(**3**)	(**4**)	(**5**)
HP-6	23.791	24.636	27.301	28.252
PPY-6	27.141	23.977	23.269	22.320
OC-6	8.397	8.207	7.239	8.176
TPR-6	19.518	11.701	14.738	13.132
JPPY-6	27.867	26.095	27.075	27.889

Table S5. Absorption maxima (cm^{-1}) in FTIR spectra of imidazole derivatives.

2-MeIm	4-MeIm	2-EtIm	2-*i*PrIm	Assignments
3136 m	3136 m	3153 w	3140 m	ν(CH) + ν(NH)
1676 m	1676 m	1673 w	1665 w	ν(C=N)
1596 vs	1596 vs	1568 s	1562 s	δ(NH) + ν(C=C) + ν(C=N)
1206 w	1206 w	1243 w	1251 w	ν(C–N) + δ(CH)
1155 vs	1155 vs	1153 m	1149 s	ν(C–C) + ν(C–N) + δ(CH)
942 s	942 s	956 s	913 s	δ(CH) + δ(Im ring)
875 w	875 w	875 w	882 m	π(CH) + δ(Im ring)
756 vs	756 vs	751 vs	751 s	π(CH)

Table S6. Absorption maxima in UV-Vis-NIR spectra of complexes (**1**)-(**5**).

Compound	Absorption maxima		Assignments
	λ [nm]	$\bar{\nu}$ [cm^{-1}]	
	215	46510	$\pi\rightarrow\pi^*$
	260	38460	$\pi\rightarrow\pi^*$
$[Cu(Macr)_2(2\text{-}MeIm)_2]$ (**1**)	335	29850	CT*
	570	17540	$d_{xz,yz} \rightarrow d_{x^2-y^2}$
	665	15040	$d_{z^2} \rightarrow d_{x^2-y^2}$
	220	45450	$\pi\rightarrow\pi^*$
$[Cu(Macr)_2(4\text{-}MeIm)_2(H_2O)]$ (**2**)	255	39215	$\pi\rightarrow\pi^*$
	665	15040	$d_{z^2} \rightarrow d_{x^2-y^2}$
$[Cu(2\text{-}EtIm)_2(Macr)_2]$ (**3**)	285	35090	$\pi\rightarrow\pi^*$
	600	16670	$d_{z^2} \rightarrow d_{x^2-y^2}$
$[Cu(2\text{-}EtIm)_2(Macr)_2]\cdot(H_2O)$ (**4**)	265	37735	$\pi\rightarrow\pi^*$
	655	15270	$d_{z^2} \rightarrow d_{x^2-y^2}$
	210	47600	$\pi\rightarrow\pi^*$
$[Cu(Macr)_2(2\text{-}i\text{PrIm})_2]\cdot CH_3OH$ (**5**)	255	39200	$\pi\rightarrow\pi^*$
	635	15750	$d_{z^2} \rightarrow d_{x^2-y^2}$

*CT= charge transfer

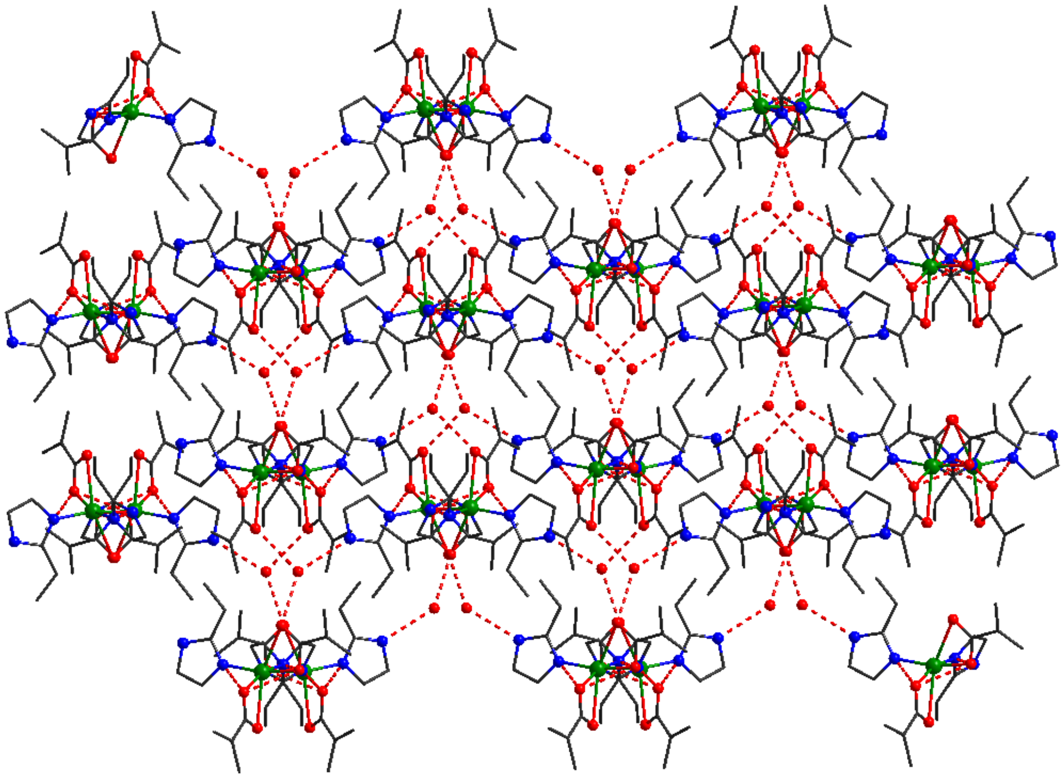

Figure S1. 3D supramolecular structure in **4**, based on hydrogen bonds interactions.

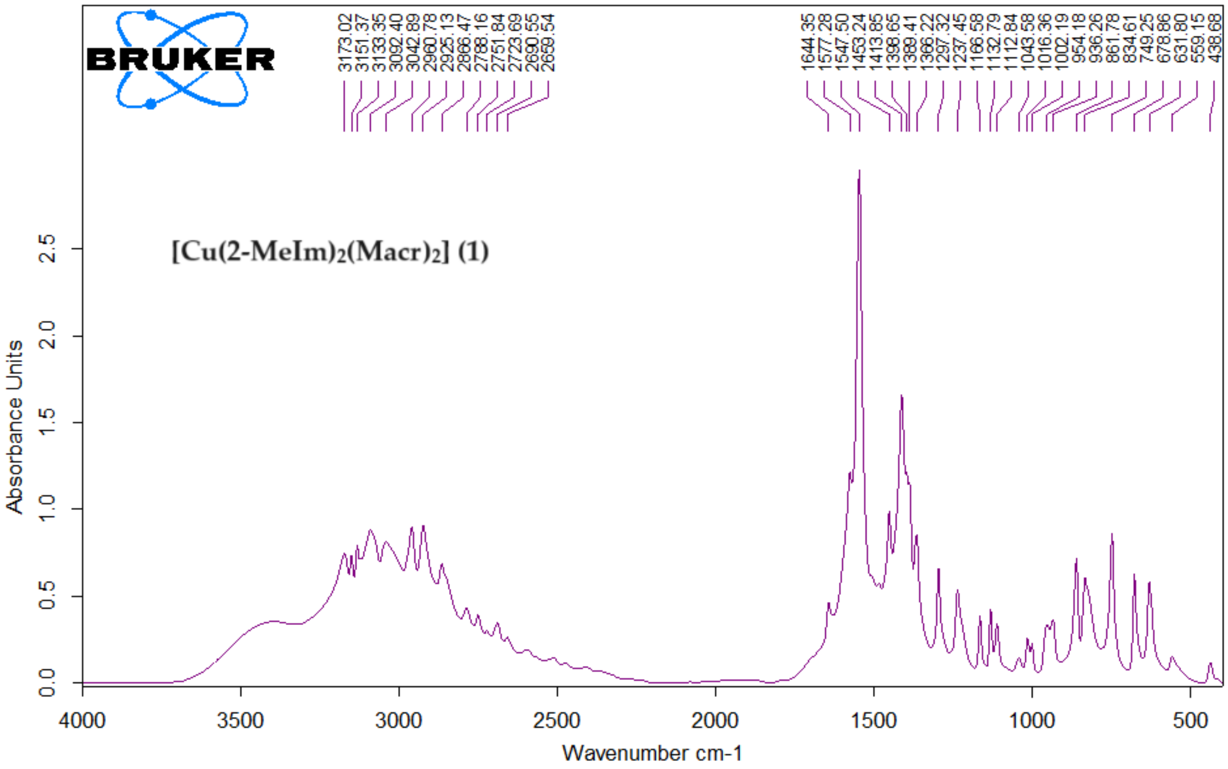

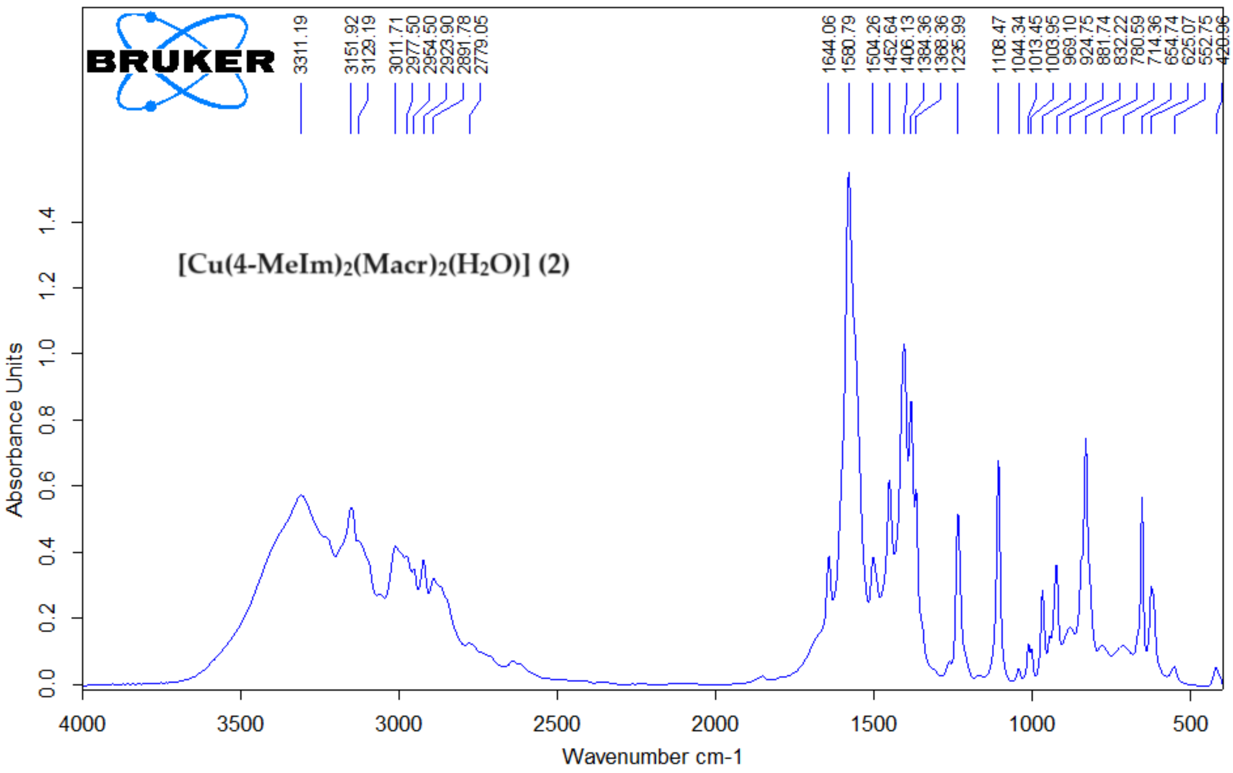
BRUKER
[Cu(4-MeIm)2(Macr)2(H2O)] (2)
Absorbance Units
Wavenumber cm-1
3311.19
3151.92
3129.19
3011.71
2977.50
2954.50
2923.90
2891.78
2779.05
1644.06
1580.79
1504.26
1452.64
1406.13
1384.36
1368.36
1235.99
1108.47
1044.34
1013.45
1003.95
969.10
924.75
881.74
832.22
780.59
714.36
654.74
625.07
552.75
420.96

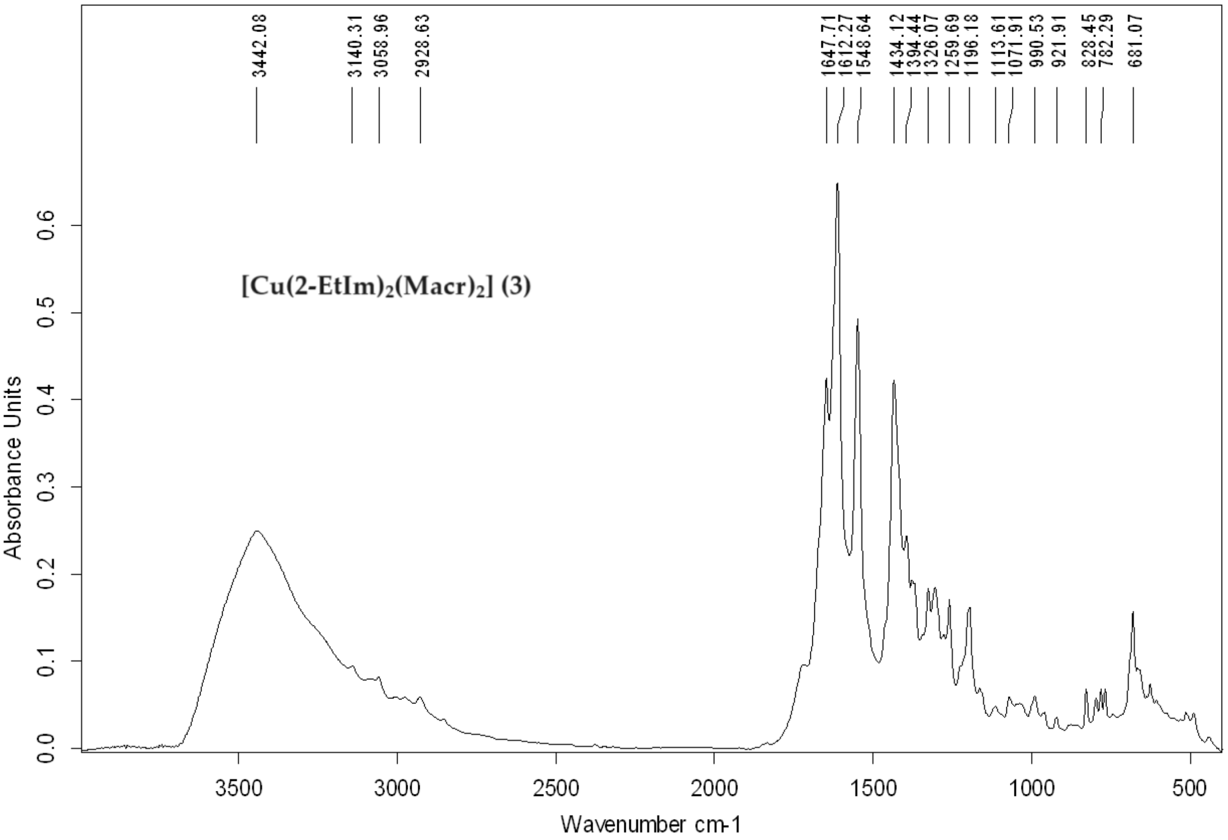
[Cu(2-EtIm)2(Macr)2] (3)
Absorbance Units
Wavenumber cm-1
3442.08
3140.31
3058.96
2928.63
1647.71
1612.27
1548.64
1434.12
1394.44
1326.07
1259.69
1196.18
1113.61
1071.91
990.53
921.91
828.45
782.29
681.07

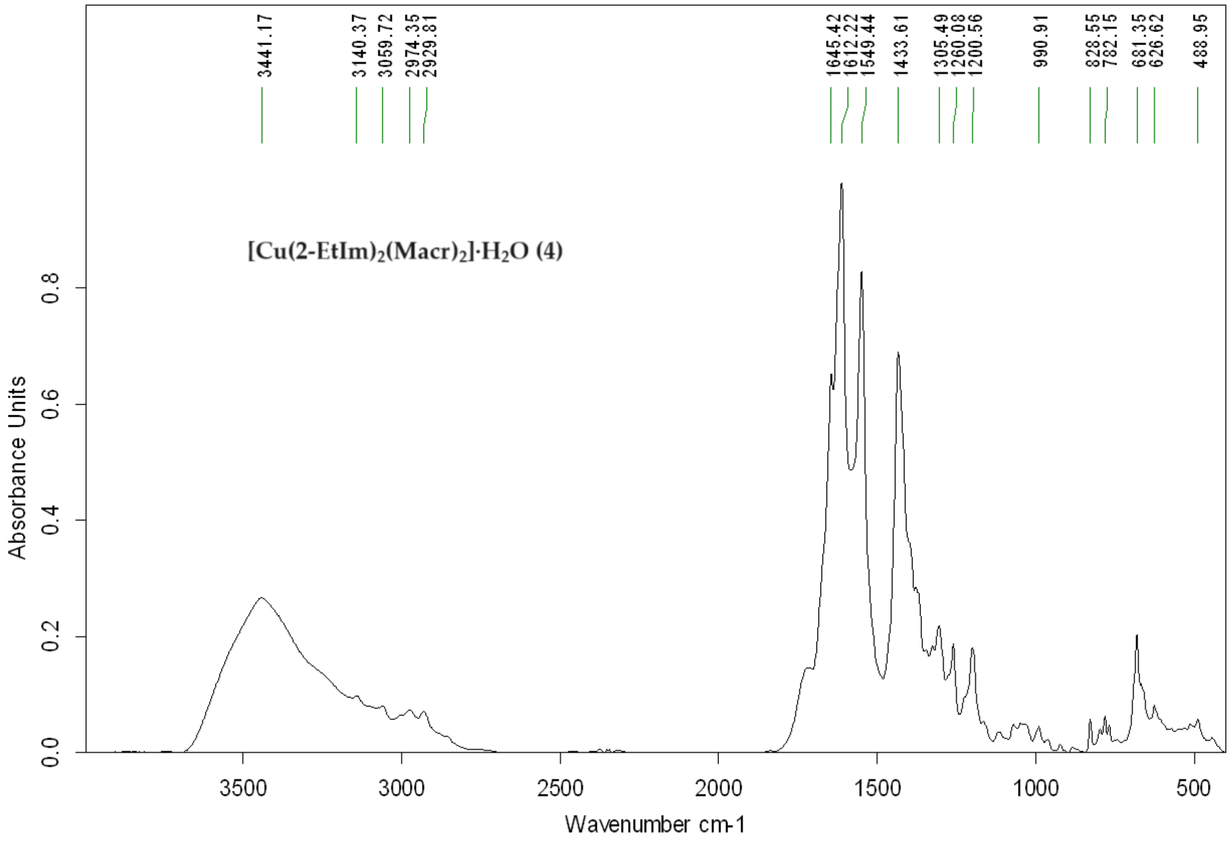

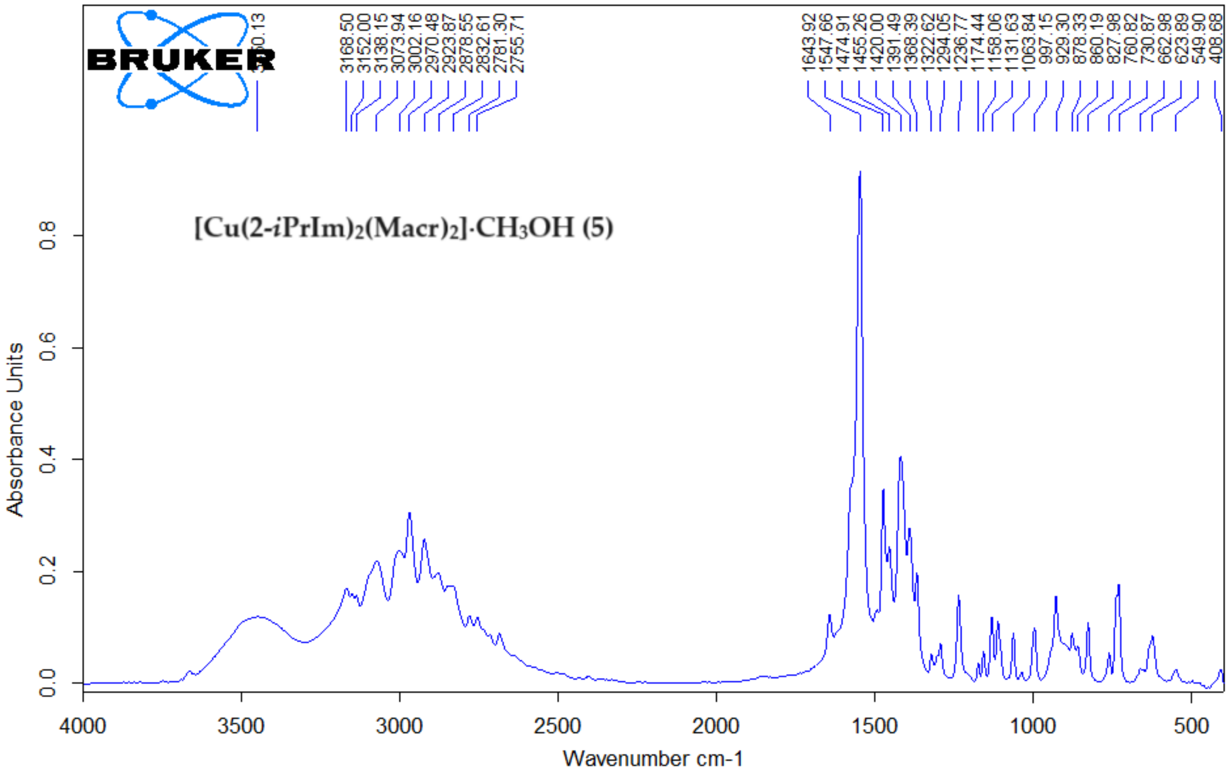

Figure S2. FTIR spectra of complexes (**1**) – (**5**).

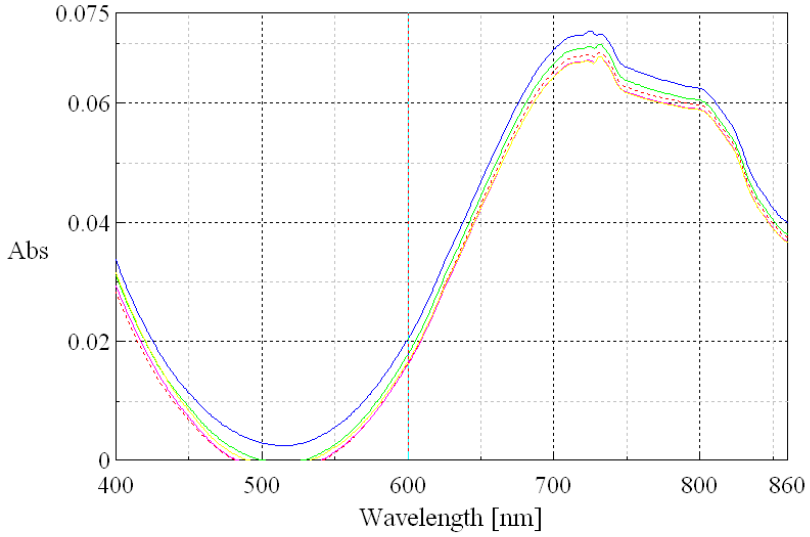
0.075
0.06
0.04
0.02
0
Abs
400
500
600
700
800
860
Wavelength [nm]

(**1**)

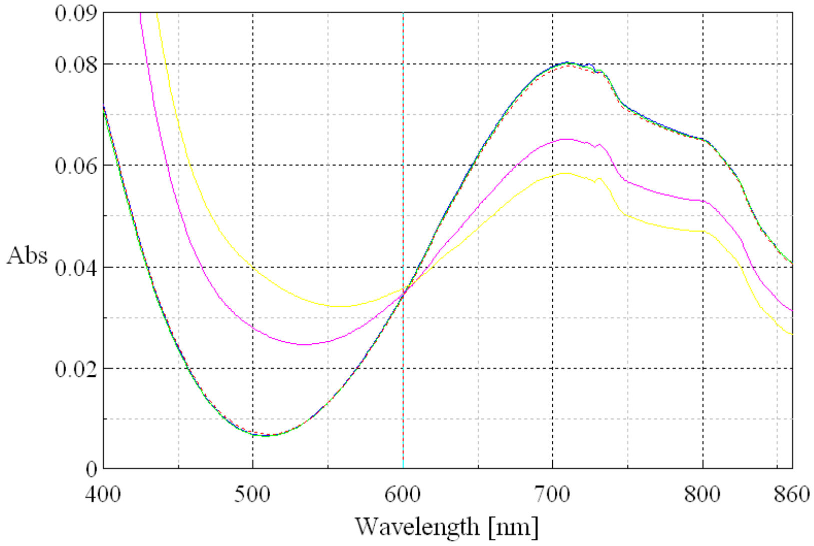
0.09
0.08
0.06
0.04
0.02
0
Abs
400
500
600
700
800
860
Wavelength [nm]

(**2**)

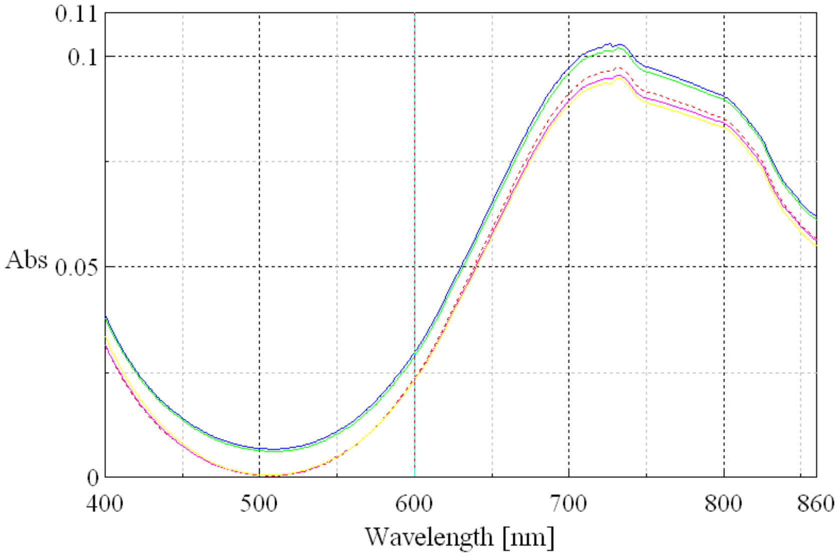
0.11
0.1
0.05
0
Abs
400
500
600
700
800
860
Wavelength [nm]

(**3**)

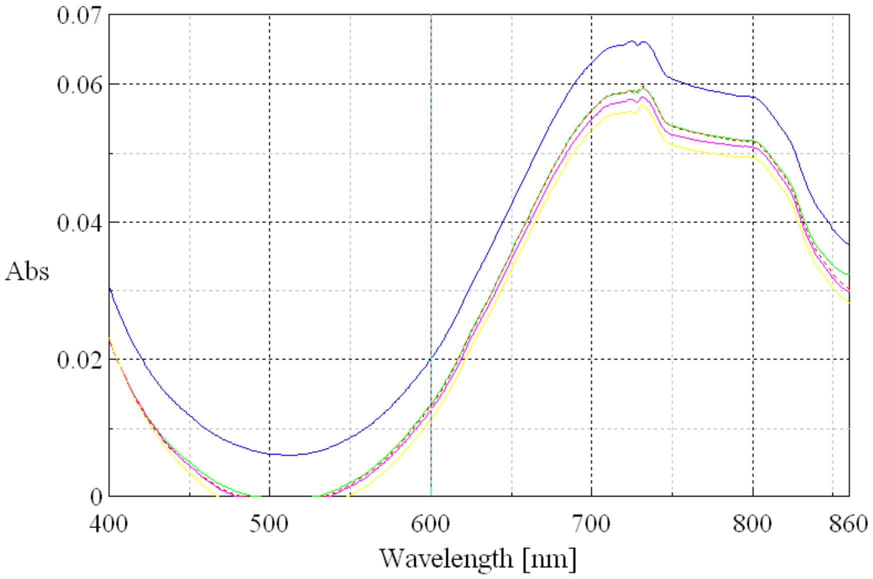

(**4**)

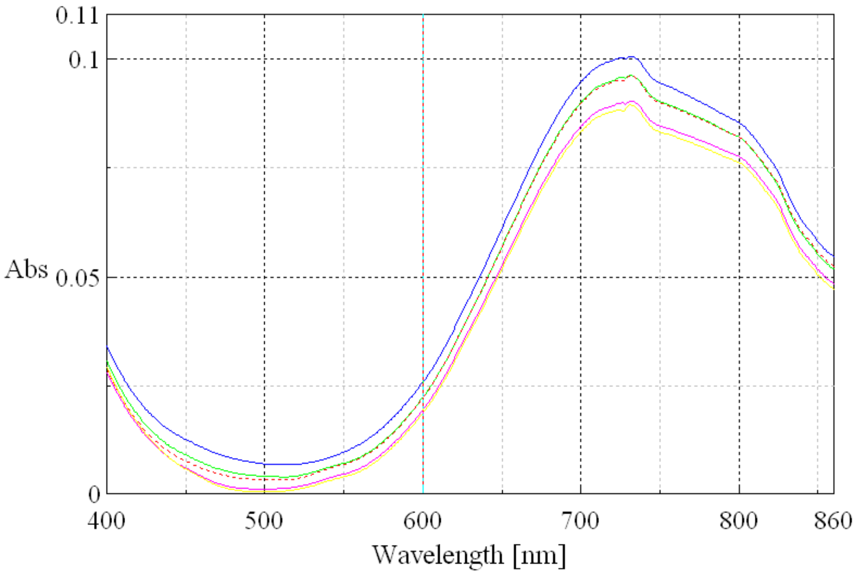

(**5**)

Figure S3. Electronic spectra in 10 μM DMSO solution of complexes (**1**)-(**5**): (blue – 0 min, green – 1 h, red – 2 h, magenta – 24 h, yellow - 48h).

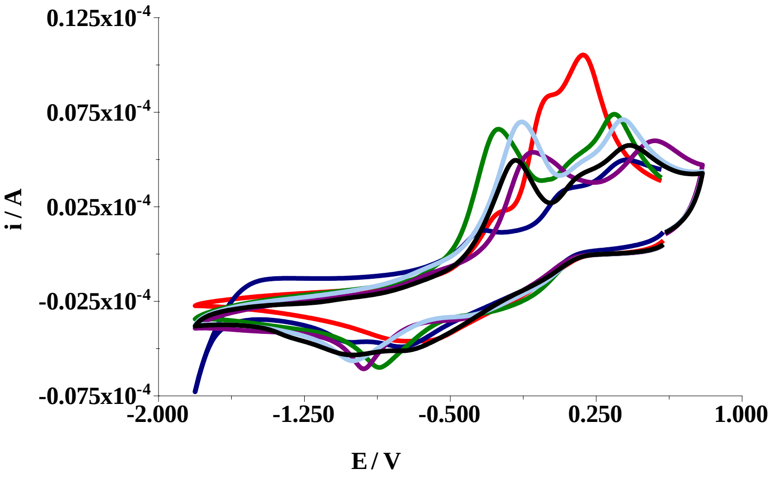

Figure S4. Cyclic voltammograms of copper complex (**1**) – (**5**); ((**1**) - (green line); (**2**) - (magenta line); (**3**) - (blue line); (**4**) - (light blue line); (**5**) - (black line)); Cyclic voltammogram for $Cu(Mcr)_2$ – red line, all concentrațions 1 mM in DMSO; supporting electrolyte - 0.1 M Bu_4NClO_4 ; scan rate: 0.050 V/s, working electrode, platinum disk, reference electrode, Ag/AgCl (0.1 M Bu_4NClO_4 in DMSO).

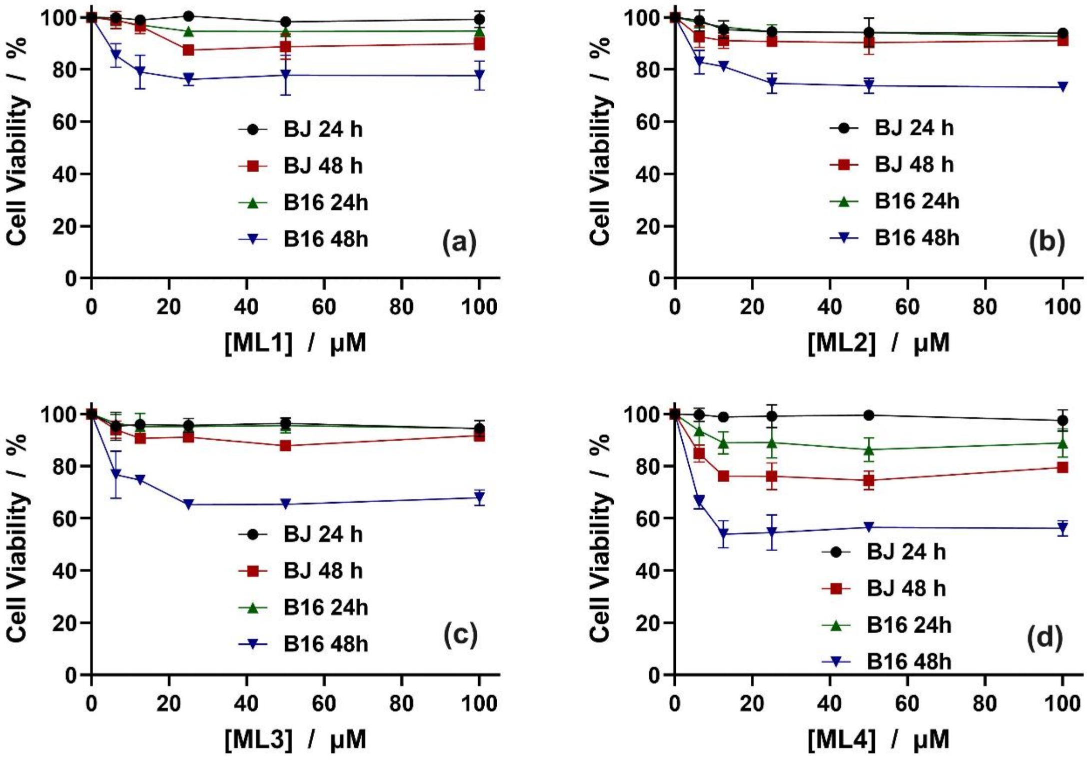

Figure S5. Cell viability of the four imidazole derivatives (ML1=2-MeIm; ML2=4-MeIm; ML3=2-EtIm; ML4=2-*i*PrIm;) against BJ and B16 cells treated for 24 and 48 h. At least three independent experiments are performed and data is represented as the means ± SD.

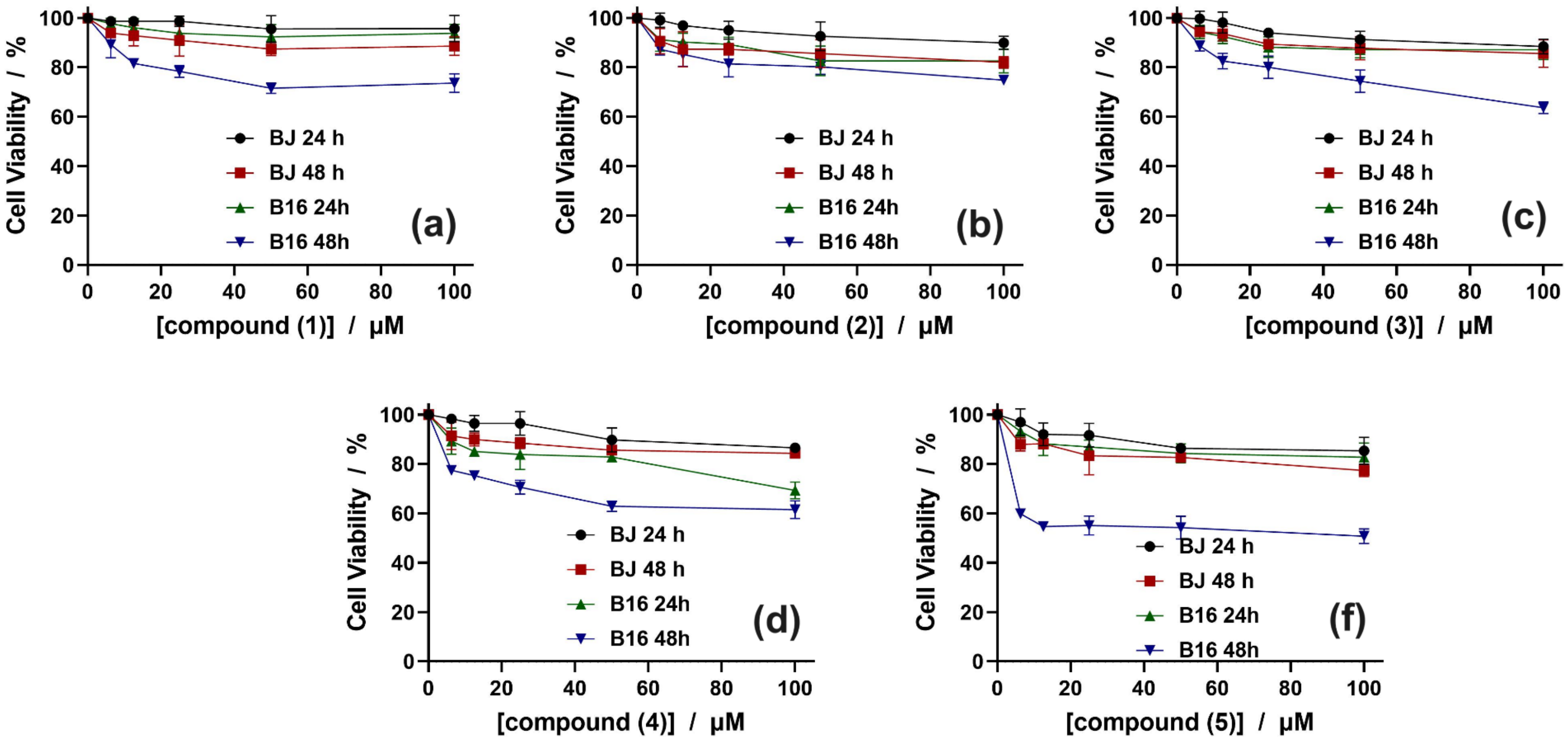

Figure S6. Cell viability of the complexes (**1**) (A), (**2**) (B), (**3**) (C), (**4**) (D) and (**5**) (F) against BJ and B16 cells treated for 24 and 48 h. At least three independent experiments are performed and data is represented as the means ± SD.
